# Understanding the Diverse Experiences of Those Living with and Beyond Cancer: Implications for Personalised Care from a Latent Profile Analysis of HRQoL

**DOI:** 10.3390/cancers17101698

**Published:** 2025-05-19

**Authors:** Laura Keaver, Christopher McLaughlin

**Affiliations:** 1 Department of Health and Nutritional Science, Atlantic Technological University, F91 YW50 Sligo, Ireland; 2 Health and Biomedical Research Centre (HEAL), Atlantic Technological University, F91 YW50 Sligo, Ireland; 3 Department of Management, Leadership & Marketing, Ulster University Business School, Belfast Campus, Belfast BT15 1AP, Northern Ireland, UK

**Keywords:** quality of life, latent profile analysis, living with and beyond cancer

## Abstract

Background and objective: Cancer and its treatments can impact HrQOL in several ways. The symptom burden both during and after treatment can be quite large in cancer patients, impacting HrQOL. In addition, changes in muscle mass can impair physical functioning, with other aspects of functioning also impacted by the cancer and its treatment. Even after treatment, physical and cognitive impairment, anxiety, and fear of cancer recurrence can persist. In clinical practice, understanding how aspects of HrQOL group together may allow clinicians to better understand and treat those living with and beyond cancer. The aim of this research was to examine the heterogeneity of HrQOL in Irish individuals living with and beyond cancer (both undergoing and completed treatment) using latent profile analysis. A secondary aim was to determine whether these groups differed by select demographic and health characteristics. Methods: Participants were recruited through the oncology day ward and outpatient department in one Irish hospital between September 2019 and March 2020. Anthropometric measures were taken and patients completed a series of questionnaires including the EORTC-QLQ-C30 to determine health-related quality of life. Latent profile analysis was undertaken using mPlus. Results: Three distinct profiles were observed: high quality of life (52.5%); compromised quality of life (34.2%); and low quality of life (13%). All groups scored lower for functioning scales (with the exception of the high quality of life group for physical, role, and emotional functioning) and higher for symptom scales then the reference norm population. There were large clinically meaningful differences between the high quality of life and low quality of life groups for all scales. The odds of belonging to the compromised quality of life group decreased significantly when having a higher handgrip strength (OR = 0.955, *p* < 0.05, CI = 0.924–0.988). Those who were working were over seven times more likely to be in the low quality of life group than the referent class (high quality of life group). The odds of belonging to the low quality of life group increased significantly for those with a higher number of nutrition impact symptoms (NISs) (OR = 1.375, *p* < 0.05, CI = 1.004–1.883). Conclusions: An empirically driven, patient-centred LPA approach characterised health-related quality of life in Irish individuals living with and beyond cancer into three distinct patient groups. This allows for more targeted, effective interventions, enhances the ability to design personalised treatment plans, and could optimise resource allocation in healthcare settings.

## Simple Summary

Cancer and its treatment can seriously affect a person’s quality of life, both physically and emotionally. This study looked at how different aspects of health-related quality of life (HrQOL) vary among those living with and beyond cancer, including those still in treatment. Researchers surveyed patients in an Irish hospital and used a statistical method to identify three distinct groups based on their HrQOL: high (52.5%), compromised (34.2%), and low (13%). People with better physical strength were less likely to have compromised HrQOL, while those with more nutrition-related symptoms or who were still working were more likely to have a lower HrQOL. All groups had more symptoms and lower functioning compared to the general population, except for the high HrQOL group. These findings highlight the need for more personalised care strategies based on a patient’s unique experiences and challenges, helping healthcare teams provide better support and use resources more effectively.

## 1. Introduction

Quality of life is a multidimensional construct that encompasses how an individual’s social, emotional, and physical traits influence everyday life. Health-related quality of life (HrQOL) can indicate how disease, disability, treatment, or a disorder can influence an individual’s well-being over time [1]. Cancer and its treatments can impact on HrQOL in several ways, such as causing fatigue, pain, emotional distress, reduced physical strength, and difficulties with memory or concentration [2].

Improvements in detection and treatments have led to improvements in survival for those diagnosed with cancer. However, it is important that these improvements in survival are accompanied by maintenance or improvements in HrQOL [3]. Symptom burden both during and after treatment can be quite large in cancer patients, impacting HrQOL [4]. In addition, changes in muscle mass can impair physical functioning, with other aspects of functioning also impacted by the cancer and its treatment [5]. Even after treatment, physical and cognitive impairment, anxiety, and fear of cancer recurrence can persist [6]. As HrQOL continues to remain lower than the general population and even other individuals with chronic disease, continued research into HrQOL in cancer survivors is important [7].

Patient-reported outcome (PRO) measures are useful at portraying the patient experience. One of the most used to determine the HrQOL is the EORTC QLQ-C30 [8]. These measures typically allocate scores for each domain (five functioning scales, eight symptom scales, and financial stress) and an overall quality of life score, which can be difficult to interpret clinically. Relying solely on overall health-related quality of life (HRQoL) scores can obscure important differences in specific functional and symptom domains among cancer survivors. Survivors with similar global HRQoL scores may report markedly different experiences—for example, one group may primarily struggle with fatigue, while another may experience significant cognitive challenges. This variability reflects underlying heterogeneity that is not captured when only mean scores are reported. Identifying distinct subgroups based on domain-specific profiles provides a more nuanced understanding of survivor well-being and may facilitate the development of more targeted and effective interventions [9].

Latent profile analysis is an empirically driven, person-centred approach that identifies latent subpopulations (individuals who are similar to each other and different from other groups/classes derived) [10]. It determines the smallest number of groups (statistically rather than subjectively) to elucidate the spread of individuals across indicators. In clinical practice, understanding how aspects of HrQOL group together may allow clinicians to better understand and treat cancer survivors. Therefore, it is important to explore HrQOL using a person-centred approach to better understand self-reported limitations and heterogeneity in those living with and beyond cancer.

The aim of this research was to examine the heterogeneity of HrQOL in Irish individuals living with and beyond cancer (both undergoing and completed treatment) using latent profile analysis. A secondary aim was to determine whether these groups differed by select demographic and health characteristics.

## 2. Materials and Methods

### 2.1. Population

Adults aged 18 years and older with a confirmed cancer diagnosis were recruited from the oncology day ward and outpatient services at Sligo University Hospital between September 2019 and March 2020. Participants completed a battery of self-report questionnaires and underwent assessments including handgrip strength testing and anthropometric measurements. Written informed consent was obtained from all participants prior to their inclusion in this study. Ethical approval was secured from the Research and Education Foundation Ethics Committee at Sligo University Hospital (Ref. No. 762).

### 2.2. Measures

#### 2.2.1. Questionnaires

Participants were asked to complete a demographic questionnaire capturing details such as gender, age, cancer type, year of diagnosis, treatments received, recent unintentional weight loss, living arrangements, educational attainment, and employment status. Nutritional status was assessed using the Patient-Generated Subjective Global Assessment short form (PG-SGA SF), a validated tool comprising four components: weight history, dietary intake, nutrition impact symptoms, and functional capacity [11]. Data on the occurrence of 13 specific nutrition impact symptoms were extracted from this instrument. The PG-SGA SF questionnaire is accessible online via the PG-SGA© | Pt-Global website. Health-related quality of life was evaluated using the European Organisation for Research and Treatment of Cancer Quality of Life Questionnaire Core 30 (EORTC QLQ-C30), focusing specifically on the Global Health Status (GHS) and five functional domains: physical, role, emotional, social, and cognitive functioning [8]. Both GHS and functional scales are scored on a 0–100 scale, with higher scores indicating a better quality of life and functioning.

#### 2.2.2. Physical Measures

Body weight and height were measured by a trained oncology nurse. Weight was assessed using a Seca column scale and recorded to the nearest 0.1 kg, while height was measured with a Seca portable stadiometer and noted to the nearest centimetre. Body mass index (BMI) was calculated using the standard formula: weight (kg) divided by height squared (m^2^). BMI classifications followed the World Health Organization (WHO) guidelines: underweight (<18.5 kg/m^2^), healthy weight (18.5–24.9 kg/m^2^), overweight (25.0–29.9 kg/m^2^), and obese (≥30.0 kg/m^2^) [12].

Isometric handgrip strength was evaluated using a spring-loaded handgrip dynamometer (Takei 5001 Hand Grip Dynamometer-Grip A, Takei Scientific Instruments Co., Ltd., Tokyo, Japan). Participants completed three trials, with each measurement recorded to the nearest 0.5 kg. The highest value among the three trials was used in subsequent analyses [13,14].

### 2.3. Latent Profile Analysis 

A three-stage approach was utilized for model selection, with latent profile analysis (LPA) being the primary method used to explore the number of latent subgroups or typologies within the data. LPA is a person-centred approach that identifies unobserved subgroups based on continuous indicators, allowing for the classification of participants based on their HRQoL responses [10]. The analysis was performed using Mplus version 6.11 [15], with robust maximum likelihood estimation applied to account for non-normality in the data. To mitigate the risk of local maxima influencing the results, 100 random sets of start values were used alongside 20 final-stage optimisations [16].

Model fit was assessed using a range of information theory-based indices, including the Akaike Information Criterion (AIC) [17], Bayesian Information Criterion (BIC) [18], and sample-size-adjusted BIC (ssaBIC) [19]. The optimal model was selected based on the lowest values for these fit indices. Additionally, the Lo–Mendell–Rubin Adjusted Likelihood Ratio Test (LMR-LRT) [20] was employed to determine the statistical significance of each successive model. A non-significant result from the LMR-LRT indicated that a more parsimonious model should be retained. Following these criteria, the model with the most meaningful and interpretable number of latent profiles was selected.

### 2.4. Multinominal Logistic Regression 

Two separate multinomial logistic regression models were generated to examine factors associated with participants’ group classifications. Model 1 assessed the influence of key sociodemographic characteristics, including age, gender, educational attainment (categorised as follows: primary/non-completed secondary; completed secondary/training; third level [e.g., BA, BSc, diploma]; and postgraduate degree or higher), and employment status. Employment was treated as a binary variable, distinguishing those not currently working (e.g., retired or unemployed) from those who were employed (full-time, part-time, or self-employed). Model 2 evaluated the relationship between classification group and selected health-related variables: body mass index (BMI, kg/m^2^), handgrip strength (kg), PG-SGA score, time since diagnosis (≤2 years vs. >2 years), and current treatment status (undergoing treatment vs. treatment completed).

## 3. Results

### 3.1. Participant Characteristics

A total of 232 participants were enrolled in this study, with a mean age of 63.5 years (SD = 11.9). The sample was predominantly female (*n* = 138, 61.1%), and most had been diagnosed within the previous five years (*n* = 167, 73.9%). Nearly half of the participants were retired at the time of this study (*n* = 112, 48.5%). The majority were actively receiving treatment (*n* = 159, 70.4%), with chemotherapy being the most commonly administered modality (*n* = 129, 81.1% of those receiving treatment), followed by hormonal therapy (*n* = 19, 11.9%). Breast cancer was the most frequently reported diagnosis (*n* = 58, 25.7%), followed by colorectal (*n* = 32, 13.8%), haematological (*n* = 28, 12.1%), lung (*n* = 12, 5.2%), and upper gastrointestinal or liver cancers (*n* = 10, 4.3%). Other cancer types included gynaecological, urinary, head and neck, skin, and bone cancers, which comprised the remainder of the cohort.

### 3.2. Fit Indices

To explore the number of QOL typologies, analysis started firstly with a one-class model and continued until models failed to add significantly to the previous model. Each of the QOL model fit indices are displayed in Table 1. A three-class model was selected as the AIC was lower in the three-class solution (AIC = 29,400.345) than the two-class solution (AIC = 29,539.776). The BIC was reported to be more favourable for the three-class model (BIC = 29,600.005) than the four-class model (BIC = 29,541.189). Additionally, since the four-class model added nothing significantly (LRT = 138.753, *p* > 0.05) to the three-class model, the three-class model was preferred. Lastly, a three-class model provides a more parsimonious explanation than a four-class model.

### 3.3. Findings from the Latent Profile Analysis

Table 2 contains the posterior probabilities for each of the three latent classes along with associated descriptive information. It also presents the group mean ± SD as well as reference values for an age-matched norm population [21] and indicates clinically relevant differences [22].

Regarding class size, it is clear from the table that the largest group is the second class (*n* = 122, 52.8%) and this group is characterised by high functioning scores and low symptom scores. Posterior probabilities ranged from 86.87 to 92.15 for functioning and from 3.25 to 19.91 for symptoms. Thus, this class of participants were labelled “high quality of life”. The next largest in participant size was the first group (*n* = 79, 34.2%), and this class was characterised by lower functioning scores than class two and the group mean, with higher burden scores than the group mean with the exception of nausea. This group was classified as “compromised quality of life” or “moderate quality of life”. Lastly, the smallest class (*n* = 30, 12.99%) was characterised by reporting lower symptom scores and higher burden scores than the other two groups and the group mean. This group was labelled “low quality of life”. 

All groups scored lower for functioning scales (with the exception of group 2 for physical, role, and emotional functioning) and higher for symptom scales then the reference norm population. There were large clinically meaningful differences between group 2 (high quality of life) and group 3 (low quality of life) for all scales. There were medium and large clinically meaningful differences between group 1 (compromised quality of life) and group 2 (high quality of life), except for appetite loss, constipation, diarrhoea, and financial, where small clinically meaningful differences were seen.

### 3.4. Findings from the Multinominal Logistic Regression

In model 1, age had only a significant effect within Class 3 (low quality of life—LQoL) (OR = 0.956, *p* < 0.05, CI = 0.917–0.998) and not Class 1 (compromised quality of life—CQoL) in comparison to the reference group (high quality of life—HQoL). Individuals in this group were slightly more likely to be younger than the reference class. Gender was significant in Class 3 (LQoL) (OR = 0.237, *p* < 0.05, CI = 0.084–0.655) and Class 1 (CQoL) (OR = 0.462, *p* < 0.05, CI = 0.246–0.869). Individuals were less likely to be female in this group in comparison to Class 2 (HQoL). Employment status was reported to have a significant effect on Class 3 (LQoL) (OR = 7.217, *p* < 0.05, CI = 1.967–26.461) in comparison to the reference class (HQoL); more specifically, workers were over seven times more likely to be in Class 3 (LQoL) than the referent class (HQoL) (Table 3).

Compared to Class 2 (HQoL) within Model 2, the odds of belonging to Class 1 (CQoL) decreased significantly when having a higher handgrip strength (OR = 0.955, *p* < 0.05, CI = 0.924–0.988). The odds of belonging to Class 3 (LQoL) increased significantly for those with a higher number of nutrition impact symptoms (NISs) (OR = 1.375, *p* < 0.05, CI = 1.004–1.883). All other associations were non-significant (Table 4).

## 4. Discussion

This study provides important insights into the heterogeneity of health-related quality of life (HRQoL) among those living with and beyond cancer, revealing three distinct HRQoL profiles: high, compromised, and low QoL. This classification supports the growing body of evidence that those living with and beyond cancer have diverse experiences with HRQoL, challenging the traditional reliance on overall scores to capture well-being. Nearly half of the participants in this study (47.2%) were classified into the compromised or low HRQoL groups, which underscores the significance of examining these subgroups more closely.

Our results also highlight the substantial impact of physical health on HRQoL, as evidenced by the significant association between handgrip strength and HRQoL. Specifically, we found that individuals with higher handgrip strength were more likely to belong to the high-quality life group, which is consistent with the existing literature emphasising the role of physical functioning in overall well-being [23]. Studies have consistently shown that reduced muscle strength and physical functioning are strongly correlated with poorer HRQoL outcomes in those with cancer [23,24,25]. For instance, a study by Esteban-Simon et al. [26] found that breast cancer survivors with greater handgrip strength and greater handgrip strength relative to their body mass index reported higher HRQoL scores across multiple domains, reinforcing the importance of maintaining physical strength during and after cancer treatment. Similarly, individuals who experience loss of muscle mass and physical deconditioning often report a higher symptom burden and lower levels of independence, which directly impacts their quality of life [27,28].

Additionally, our study found that participants with higher numbers of nutrition impact symptoms (NISs) were more likely to belong to the low QoL group. This is particularly noteworthy given that NISs, such as loss of appetite, nausea, and digestive issues, are common among those living with and beyond cancer and are well-documented in the literature as having a detrimental effect on QoL [29]. As cancer treatments can often lead to gastrointestinal disturbances and a reduced food intake [30], the impact of NISs on physical function, mental health, and overall quality of life cannot be understated. Nutrition-related symptoms can contribute to increased fatigue, reduced physical function, and compromised emotional well-being [31], highlighting the importance of comprehensive nutritional assessment and interventions as part of cancer care to mitigate the negative impact of NISs.

Our findings also shed light on the influence of employment status on HRQoL. Specifically, we found that individuals who were employed at the time of the study were over seven times more likely to belong to the low QoL group than those who were retired or unemployed. This finding raises important questions about the challenges that individuals with cancer face when attempting to balance their recovery with the demands of work. Physical and psychological burdens can be particularly taxing for individuals trying to maintain a full-time job or return to work after treatment [32].

These findings highlight the necessity of tailored, individualised interventions that address the specific needs of those living with and beyond cancer based on their unique HRQoL profiles. Those living with and beyond cancer face a wide range of challenges, and it is crucial that clinicians recognise and address the variability in HRQoL to provide optimal care. For example, interventions aimed at improving physical function, such as exercise programmes, could be particularly beneficial for individuals in the compromised or low HRQoL groups, as physical deconditioning is a key determinant of poorer outcomes [27]. Similarly, targeted nutritional support is vital for those experiencing NISs, as managing symptoms like nausea and appetite loss can significantly improve physical health and overall well-being. Furthermore, policies and workplace accommodations should be considered to help survivors maintain or return to work without exacerbating their physical or psychological burdens, ultimately improving their QoL and long-term recovery.

Our study suggests that HRQoL interventions should not be one-size-fits-all but rather tailored to the needs of each individual. The identification of distinct HRQoL profiles, such as those seen in this study, presents an opportunity to personalise care strategies and enhance the effectiveness of interventions. For example, those in the low QoL group may benefit from more intensive physical rehabilitation and psychosocial support, whereas those in the high QoL group may require less frequent follow-up and interventions. Implementing personalised care plans, along with routine screening for symptoms of physical deconditioning, nutrition issues, and mental health concerns, can improve the overall experience and help individuals achieve a higher quality of life post-treatment.

While the present study focused on individual HrQOL profiles among those living with and beyond cancer, it is important to recognise the broader social context. The existing research highlights the significant role that family members play in influencing a patient’s psychosocial and emotional well-being [33]. Future research could usefully explore how family functioning and caregiver dynamics interact with latent HrQOL profiles. Identifying such interactions may inform the development of more holistic, patient-centred interventions that incorporate family or caregiver support as a component of care.

## 5. Conclusions

This study identified three distinct health-related quality of life (HRQoL) profiles among those living with and beyond cancer using a person-centred latent profile analysis approach. These profiles—high, compromised, and low quality of life—highlight substantial heterogeneity in individual experiences that is not captured by average HRQoL scores alone. Significant differences across the profiles were associated with key demographic and clinical factors, including handgrip strength, employment status, and the presence of nutrition impact symptoms. These findings underscore the importance of adopting nuanced, stratified approaches in cancer care. Recognising and responding to subgroup-specific needs may enhance the effectiveness of interventions, inform personalised care planning, and support a more efficient allocation of healthcare resources. Future research should explore how these profiles evolve over time and whether targeted interventions can improve outcomes within lower HRQoL subgroups.

## Figures and Tables

**Table 1:** Latent profile fit indices for two- to five-class solutions.

Class	Loglikelihood	Par	AIC	BIC	Adj LRT	*p*
1	−15,064.791	28	30,185.581	30,281.969	--	--
2	−14,726.888	43	29,539.776	29,687.800	667.627	*p* < 0.001
**3**	**−14,642.172**	**58**	**29,400.345**	**29,600.005**	**167.381**	***p* < 0.05**
4	−14,571.946	73	29,289.892	29,541.189	138.753	ns
5	−14,480.557	88	29,137.113	29,440.046	180.567	ns

Note: Par = Number of Parameters; AIC = Akaike Information Criterion; BIC = Bayesian Information Criterion; LRT = Lo–Mendell–Rubin likelihood ratio test; ns = not significant. Best-fitting LCA model in bold.

**Table 2:** Descriptive information regarding the three classes that arose from LPA *.

	Class 1	Class 2	Class 3	Group Mean ± SD	Norm-Population Mean [15]	Clinically Meaningful Differences [16]			
						Trivial	Small	Medium	Large
**Physical Functioning**	**71.29**	**91.81**	**49.53**	**79.31 ± 20.02**	89	0–5	5–14	14–22	>22
Role Functioning	64.95	**92.15**	37.45	75.75 ± 29.39	88	0–6	6–19	19–29	>29
Emotional Functioning	76.06	**90.83**	49.98	80.53 ± 22.66	90	0–3	3–7	7–10	>10
Cognitive Functioning	77.85	**89.25**	53.13	80.66 ± 23.29	92	0–3	3–9	9–14	>14
Social Functioning	66.65	**86.87**	36.69	73.44 ± 28.10	95	0–5	5–11	11–15	>15
Fatigue	**39.52**	14.11	**75.35**	30.82 ± 26.20	5	0–5	5–13	13–19	>19
Nausea/Vomiting	7.70	3.25	**34.20**	8.80 ± 19.08	1.8	0–3	3–8	8–15	>15
Pain	**22.19**	6.18	**61.37**	18.83 ± 28.36	18	0–6	6–13	13–19	>19
Dyspnoea	**24.89**	9.84	**40.83**	19.03 ± 27.04	7.4	0–4	4–9	9–15	>15
Sleep Disturbances	**37.62**	19.91	**67.85**	32.17 ± 37.10	5	0–4	4–13	13–24	>24
Appetite Loss	**16.56**	6.12	**44.77**	14.72 ± 27.71	3	0–5	5–14	14–23	>23
Constipation	**15.28**	10.75	**31.57**	15.00 ± 27.38	7.7	0–5	5–13	13–19	>19
Diarrhoea	**12.59**	7.33	**29.21**	11.98 ± 24.78	4.2	0–3	3–7	>7	-
Financial	**18.61**	11.25	**38.12**	17.17 ± 26.78	2.1	0–3	3–10	>10	-
**N**	79	122	30						
**%**	34.20	52.81	12.99						

Note: Probabilities greater than the mean for this cohort for each of the QoL domains are bolded. For functioning scales higher numbers indicate greater functioning, for symptom scales higher numbers indicate greater burden. * 231 were successfully classified by LPA.

**Table 3:** Odds ratios and confidence intervals (95%) for demographic factors.

Class 2 (HQoL)		SE	OR		95% Confidence Interval	
					Lower	Upper
Class 1 (CQoL)	Age	0.017	1.012		0.980	1.046
	Education	0.202	0.968		0.651	1.439
	BMI (kg/m^2^)	0.026	1.010		0.960	1.063
	Gender	0.322	0.462	*	0.246	0.869
	Male = 0, Female = 1					
	Work	0.369	1.171		0.568	2.415
	Not working = 0, Working = 1					
Class 3 (LQoL)	Age	0.022	0.956	*	0.917	0.998
	Education	0.314	0.676		0.365	1.250
	BMI (kg/m^2^)	0.033	1.034		0.969	1.103
	Gender	0.526	0.237	*	0.084	0.665
	Male = 0, Female = 1					
	Work	0.663	7.215	*	1.967	26.461
	Not working = 0, Working = 1					
The reference category is Class 2.						

Note: SE = standard error, OR = odds ratio * = *p* < 0.05.

**Table 4:** Odds ratios and confidence intervals (95%) for health-related factors.

Class 2 (HQoL)		SE	OR		95% Confidence Interval	
					Lower	Upper
Class 1 (CQoL)	Handgrip (kg)	0.017	0.955	*	0.924	0.988
	Total NISs	0.135	1.266		0.972	1.650
	PG-SGA	0.380	1.098		0.521	2.315
	Diagnosed within 2 years	0.338	1.145		0.590	2.223
	No = 0, Yes = 1					
	Receiving treatment	0.368	0.778		0.378	1.602
	No = 0, Yes = 1					
Class 3 (LQoL)	Handgrip (kg)	0.028	0.962		0.911	1.016
	Total NISs	0.160	1.375	*	1.004	1.883
	PG-SGA	0.527	2.363		0.842	6.632
	Diagnosed within 2 years	0.509	2.253		0.831	6.109
	No = 0, Yes = 1					
	Receiving treatment	0.548	0.819		0.280	2.394
	No = 0, Yes = 1					
The reference category is Class 2.						

Note: SE = standard error, OR = odds ratio, * = *p* < 0.05.

## Data Availability

The datasets generated during and/or analysed during the current study are available from the corresponding author on reasonable request.
